# The emerging landscape of circular RNAs in immunity: breakthroughs and challenges

**DOI:** 10.1186/s40364-020-00204-5

**Published:** 2020-07-10

**Authors:** Zhouxiao Li, Ye Cheng, Fan Wu, Liangliang Wu, Hongyong Cao, Qian Wang, Weiwei Tang

**Affiliations:** 1 Department of Hand Surgery, Plastic Surgery and Aesthetic Surgery, Ludwig-Maximilians University, Munich, Germany; 2 Department of General Surgery, Nanjing First Hospital, Nanjing Medical University, Nanjing, Jiangsu China

**Keywords:** circRNAs, Immunity;T lymphocytes;macrophages;infection

## Abstract

Circular RNAs (circRNAs) are covalently linked RNAs that exhibit individual strand with a closed-loop framework compared with a conserving, steady and abundant linear counterpart. In recent years, as high-throughput sequencing advancement has been developing, functional circRNAs have been increasingly recognized, and more extensive analyses expounded their effect on different diseases. However, the study on the function of circRNAs in the immune system remains insufficient. This study discusses the basic principles of circRNAs regulation and the systems involved in physiology-related and pathology-related processes. The effect of circRNAs on immune regulation is elucidated. The ongoing development of circRNAs and basic immunology has multiplied their potential in treating diseases. Such perspective will summarize the status and effect of circRNAs on various immune cells in cancer, autoimmune diseases and infections. Moreover, this study will primarily expound the system of circRNAs in T lymphocytes, macrophages and other immune cells, which creates a novel perspective and lay a theoretical basis for treating diseases.

## Introduction

Circular RNAs (circRNAs) are an emerging RNAs type differing from traditional linear RNAs. They are abundant in eukaryotic transcriptome and form covalently closed continuous loops, where the 3′ and 5′ ends presenting in an RNA molecule in a normal manner are joined jointly [1]. Such characteristic induces the property of circRNAs, covering microRNA(miRNA) sponge, showing an interaction to RNA-binding proteins (RBPs), and encoding proteins that has been recently identified [2]. CircRNAs are found to impact considerable physiological and pathology processes, covering regulated cell death, metabolism, cancer, as well as drug resistance [3–5]. Besides, circRNAs can be up-regulated and stable in exosomes, which has increased opportunities for intercellular communication [6]. The dysregulation of circRNA expression is closely associated with the occurrence of a wide range of diseases in human beings. Indeed, the current research and reports of circRNAs in the field of cancer are the most.

The immune system accounts for maintenance of internal homeostasis by immune regulations, by monitoring and preventing the invasiveness of pathogens. The immune response of the synthesis of multiple immune cells elicits antiviral, antibacterial and antitumor functions. Though existing researchers have more focused on proteins, numerous researches suggested that noncoding RNAs may also be considered novel candidates helping regulate immune diseases and responses [7, 8]. It has been newly evidenced that circRNAs participate in immune responses, though their effect remains unclear. Here, we primarily elucidated the status and effect of circRNAs on a wide variety of types of immune cells in cancer, autoimmune diseases, and infections, which can present a novel perspective and lay a theoretical foundation in treating diseases.

## Biological functions of circRNAs

Recently conducted researches indicated that circRNAs can be used as a miRNA sponge to inhibit targeted mRNA functions, showing interaction to RNA-binding proteins (RBPs) and translating proteins [9]. Among them, the functional effect of miRNA sponge is the most extensively known. However, binding to other proteins and translating proteins have established novel directions to study circRNAs.

### MiRNA sponge

**Fig. 1:**
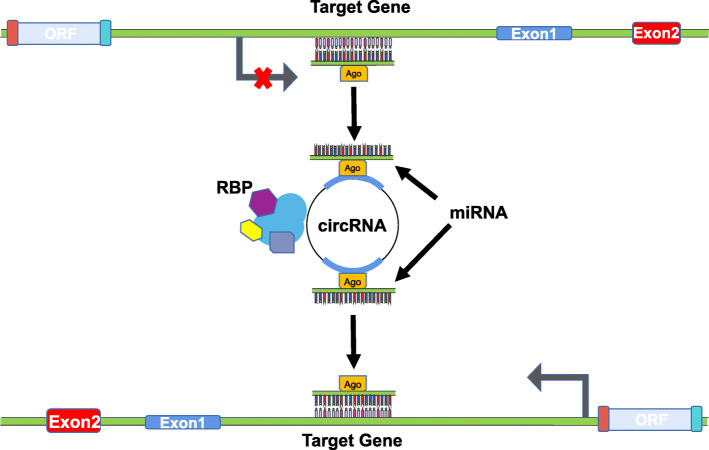
CircRNAs can reduce the ability of miRNAs to bind to target mRNAs by acting as a binding platform for Ago2 and miRNAs and interact with RBPs

MiRNAs are small (~ 21 nt) non-coding RNAs identified in some viruses, animals and plants, inhibiting translation of messenger RNAs that participate in significant and different biology-related processes. For the presence of different binding sites recognizing a seed region, circRNAs can sponge up a family’s miRNAs, thus becoming relatively efficient inhibiting elements and then releasing target mRNAs [10]. The Argonaute protein (Ago) family are the “effector proteins” that promote miRNAs to fulfill the effects, as well as being the core factors of RNA silencing. They can bind to a range of small non-coding RNAs categorizes (covering miRNAs) and get involved in the suppressing process for mRNA cleavage or translating process [11]. It has been reported that many circRNAs reduce the ability of miRNAs by binding to target mRNA via being a binding basis in terms of Ago2 and miRNA (Fig. 1).

### Interacting with RBPs

RBPs are proteins undertaking gene transcribing and translating processes together with circRNAs and impact circRNA processing, folding, and localization [12] (Fig. 1). For instance, in *Drosophila melanogaster*, Mbl protein can promote the formation of circRNA by binding to introns on the exon flanks [13]. During epithelial-mesenchymal transition, under the relatively high expression of QKI protein, mRNA is formed, and the highly expressed QKI protein is capable of binding to the intron flanking the exon, making the exons side by side to promote cyclization [14]. In contrast to the effect of QKI protein, high expression of ADAR1 protein can inhibit the formation of circRNA by breaking the RNA pairing of exon flanks [15].

### Translation of proteins and peptides

**Fig. 2:**
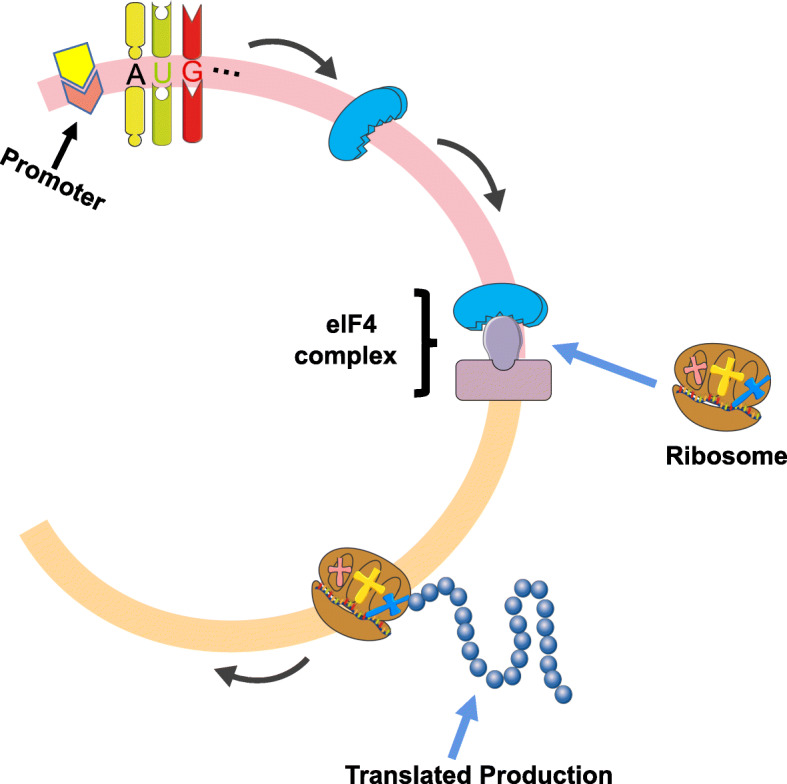
Translation of circRNAs: a circRNA containing an open reading frame (ORF) driven by the internal ribosome entry site (IRES) can translate a functional protein

CircRNAs were originally defined as non-coding RNAs. As fueled by the efficient advancement of bioinformatics analysis and high-throughput sequencing techniques, some circRNAs have been found to be able to translate proteins and peptides. CircRNAs covering an open reading frame (ORF) stimulated through an inner ribosome entry site (IRES) have the potential to translate proteins (Fig. 2) [16]. For instance, circZNF609 effectively indicates circRNA translation and covers an ORF spanning the initiation codon and terminating in the in-frame stop codon, so cyclization complies with linear transcript [17]. Moreover, as the most affluent RNA modification in eukaryotes, N6-methyl adenosine (m6A) has been suggested to be closely associated with circRNA mediated protein translation as well. Yang Y et al. reported a recognized m6A motif enrichment on circRNAs, and a single m6A site was found sufficient to initiate translating process. Such translation initiated by m6A required the initiation factors eIF4G2 and m6A recognition protein YTHDF3, as enhanced by methyltransferase METTL3/14, hindered by demethylase FTO, and enriched by heat shock [18]. The mentioned outcomes present a novel insight into circRNAs functions in physiological and pathological processes, which also changes the original concept that circRNAs do not participate in protein coding.

## Physiology and pathology-related processes with circRNAs involved in

### circRNAs can regulate regulated cell death (RCD)

RCD was employed for expressing the death of cells originating from the intracellular or extracellular microenvironment performed by molecular systems when other adaptive responses cannot restore cell homeostasis, which, according to their different systems, can fall into apoptosis, autophagic cell death, ferroptosis, etc. [19, 20] It has been reported that circRNA is capable of regulating RCD especially autophagy and ferroptosis. Autophagy is a mature and conserved system delivering intracellular components and organelles to lysosomes for degradation process [21]. Disorders of autophagy are associated with considerable diseases. Emerging studies revealed a novel circRNA termed as autophagy-associated circular RNA (ACR) to regulate autophagy. This study showed that ACR protected the heart from ischemia/reperfusion injury and narrowed myocardial infarction area. ACR activated Pink1 expressing process by directly binding to Dnmt3B and blocking DNA methylation of Dnmt3B mediated Pink1 promoting element, thus inhibiting autophagy [22]. Ferroptosis has been defined recently as a non-apoptotic, RCD procedure covering the abnormal metabolism of lipid oxides in cells catalyzed by iron ions or iron enzymes [23]. In such process, a wide range of inducers break the cell redox balance and produce considerable lipid peroxidation products, thus triggering cell death. Zhang HY et al. reported that circ-TTBK2 knockdown or miR-761 increase could retard cell proliferation, invasion, and promote ferroptosis in glioma cells [24]. The above data shows that the effect of circRNAs on RCD requires subsequent exploration, and more specific investigation is required in this aspect, opening more prospects for ongoing and future research.

### circRNAs is involved in cell metabolism

Metabolism Resetting of energy is a hallmark of tumors attributed to genome instable state. According to recently conducted researches, circRNAs participate in metabolism covering glycolysis, fatty acid metabolism, and amino acid metabolism. Li Q et al. proved that circMAT2B enriched PKM2 by sponging miR-338-3p, which encoded a vital enzyme during glycolysis and facilitated hepatocellular carcinoma (HCC) progression [25]. Li H’s team identified that circ-CUX1 bonded to EWS RNA-binding protein 1 (EWSR1) to expedite its interacting process with MYC-related zinc finger protein (MAZ), thus leading to promotion of aerobic glycolysis and tumor progression in neuroblastoma [26]. An inverse association between circ_0046366 expressing and triglyceride (TG) level in HepG2 cell culture and liver tissues was identified [27]. Circ_0046366 could sponge miR-34a to protect receptor (PPAR) α stimulated by the peroxisome proliferator from transcriptional repression. PPARα activated CPT2 and ACBD3 to degrade lipids. Researches also delved into the effect of circRNAs on glutamine metabolism and identified the circ_002581/miR-122/Slc1a5 axis in non-alcoholic steatohepatitis [28].

### circRNAs are enriched and stable in exosomes

Exosomes are endocytic origin’s small membrane vesicles enegrated by majority of cells. They cover species of proteins, mRNAs and miRNAs that regulate the behaviors of recipient cells and become biomarkers for the diagnosis of human diseases [29]. Li Y et al. in 2015 first confirmed the existence of considerable circRNAs in exosomes [30]. Next, the association between circRNAs and exosomes began to rise. RNA-seq analysis was conducted for detecting the abundance of circRNAs in exosomes from serum and follicular fluid. Note that Wang G et al. reported that higher metastatic HCC endowed potential with less or no metastatic potential by exosomes covering circ-PTGR1, thereby leading to stronger migration and invasion of tumor cells [31]. Likewise, exosomal circRNAs secreted by adipocytes have been reported for facilitating tumor development and mitigating DNA impairment via hindering miR-34a and stimulating USP7/cyclin A2 signaling path [32].

### circRNAs and drug resistance

Though existing targeted drugs perform well in malignancy treatment, drug resistance is still inevitable. Therefore, it is extremely crucial to deeply understand the drug resistance system and find new therapeutic target. Xu N et al. employed high-throughput circRNA chips to detect the A549-sensitive strain and paclitaxel-resistant strain A549 / Taxol. In contrast to the sensitive strains, the expression of 2909 circRNAs in A549 / Taxol was noticeably enriched and 8372 circRNAs were noticeably declined, demonstrating that abnormal circRNA is likely to alter the occurrence of paclitaxel resistance [33]**.**Circ-PVT1 was found to facilitate paclitaxel resistance of gastric cancer cells by controlling ZEB1 expressing via the sponging process for miR-124-3p [34].

### circRNAs and cancer

**Fig. 3:**
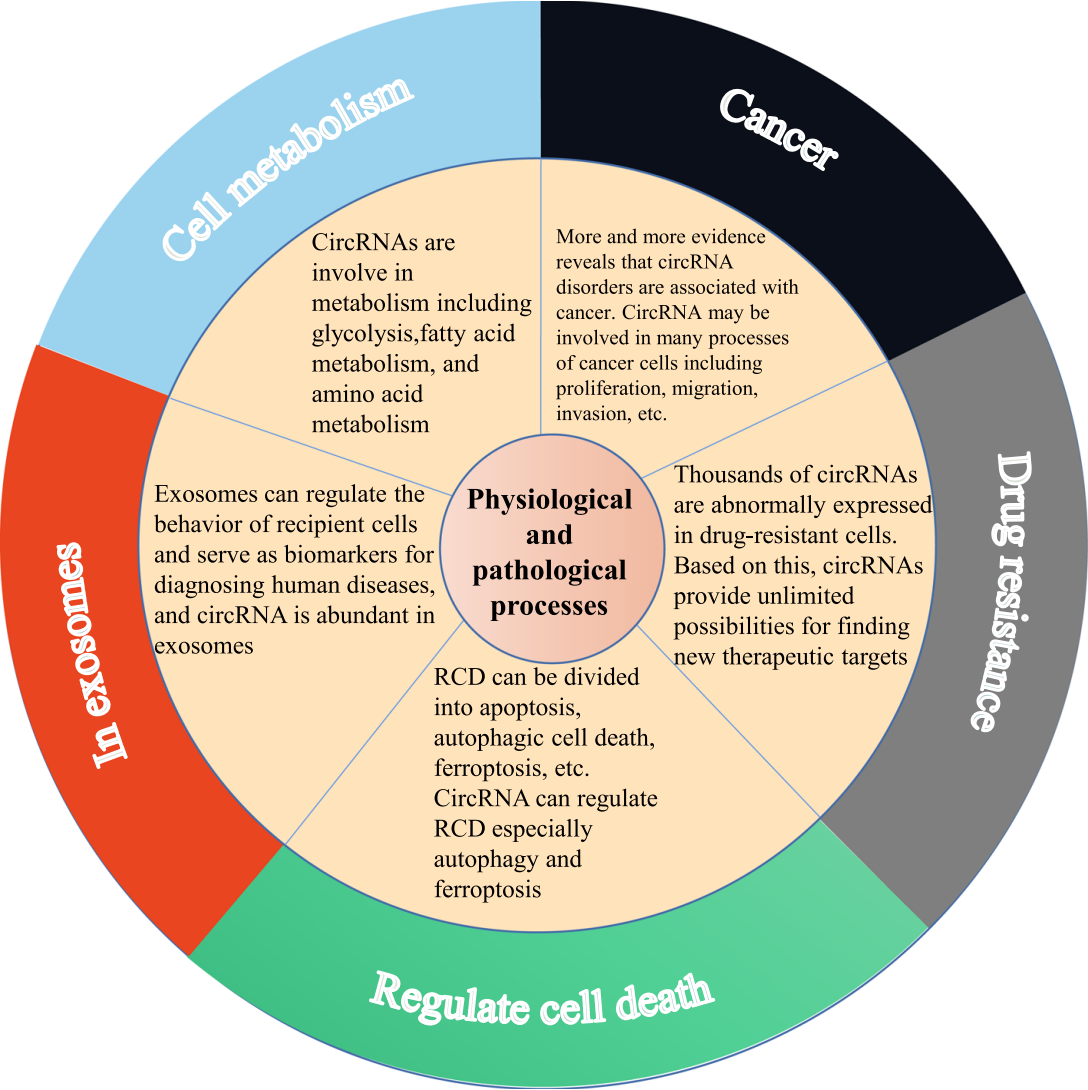
Physiological and pathological processes that circRNAs involve in

Note that there are numerous causes of circRNA disorders in cancer (e.g., aberrant cis-elements, aberrant chromosomes and genomes, aberrant transcription, aberrant spliceosomal machinery, and aberrant trans-acting elements) [35]. Liu W et al. illustrated a novel circ_103809 /miR-4302/ZNF121/MYC regulating signaling pathway promotes lung cancer progression [36].Bian LJ et al. and Zhang PL et al. indicated that circ_103809 may be a potential novel gene target for the diagnosis and treatment of colorectal cancer(CRC) by controlling biological functions via the miR-532-3P/FOXO4 axis [37, 38].Song LL et al. suggested an oncogenic role for circ_0007534 in breast cancer through being a miR-593 sponge for enriching MUC19 expression [39] .However, the studies on circRNAs in cancer recurrence and metastasis remain unmatured, and further exploration is required. In this content, a figure was generated for illustrating the angle and direction of circRNA when taking part in physiology- and pathology-related processes (Fig. 3).

### Circular RNAs in immune responses and diseases

**Fig. 4:**
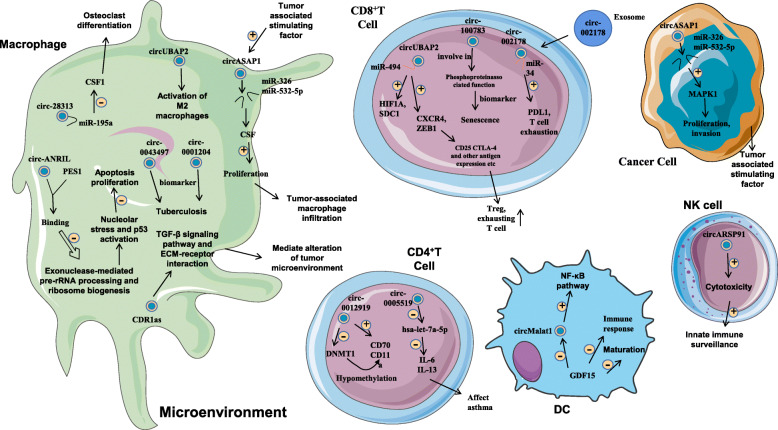
Correlation and regulatory mechanisms of circRNAs in immune responses and diseases. CircRNAs regulate immune response and participate in the occurrence and development of diseases through direct regulation mechanism or ceRNA mechanism. CSF:Macrophage colony-stimulating factor. PES1:Pescadillo homologue 1. DNMT1:DNA methyltransferase 1. GDF15:Growth/differentiation factor 15. MAPK1:Mitogen-activated protein kinase 1. CXCR4:C-X-C chemokine receptor type 4. ZEB1:Zinc finger E-box-binding homeobox 1. CTLA-4:Cytotoxic T-lymphocyte protein 4

**Fig. 5:**
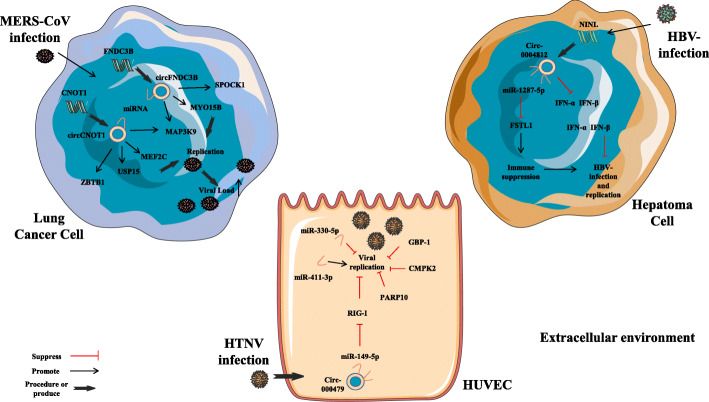
CircRNA and viral infection. CircRNAs act as miRNA sponge to regulate downstream molecules, which affects virus replication and immune microenvironment, producing promote or inhibit effect in viral activity. MAP3K9:Mitogen-activated protein kinase kinase kinase 9. RIG-I:Retinoic acid inducible gene I. FSTL1:Follistatin-related protein 1. IFN:Interferon

The immune system should monitor and defend against a wide variety of exogenous pathogens, and it should maintain internal homeostasis by maintaining appropriate immune tolerance and regulation. In fact, immune surveillance and defense act as the major immune responses. Based on the type of foreign antigen, the immune response can perform antiviral, antibacterial, and anti-tumor functions, depending on the synthesis of a wide range of immune cells that build the immune defense [40–43]. Factors (e.g., improper exposure to autoantigen, maladjustment of immune response and cross-antigen stimulation) may induce autoimmunity and facilitate the progress of several immune diseases [44–46]. Immunity can fall into innate and adaptive immunity. Innate immunity is defined as the first line of host defense against pathogens and gives rise to adaptive immune system to conduct effector functions. The innate immune system is primarily mediated by dendritic cells (DCs), macrophages, and natural killer cells (NKs). A response by innate immunity is induced for the pathogen that gives rise to an antigen-specific adaptive immune response. Adaptive immunity exhibits high specificity and covers primarily T and B lymphocytes. CircRNAs were found to facilitate immune responses and impact the processes of autoimmune diseases, tumor immunity and antiviral immunity. In this study, the correlation and regulating systems of circRNAs in a wide variety of immune responses and diseases are summarized (Fig. 4, 5, Table 1) [47–84] .

**Table 1:** circular RNAs in immune responses and diseases

circRNA	Immune cell types	Potential Functions	Diseases or Process	Reference
circ_002178	CD8^+^ T cell	acting as a ceRNA to promote PDL1/PD1 expression	LUAD	[47]
circ_100783	CD8^+^ T cell	playing a role in phosphoprotein-associated functions duringCD28-related CD8^+^ T cell ageing	–	[48]
circ-TNFRSF11A	CD4^+^ T cell	participating in the SCID mediated alteration of different signaling pathways.	SCID	[49]
circ_0005519	CD4^+^ T cell	inducing IL-13 and IL-6 expression by regulating let-7a-5p in CD4^+^ T cells to affect asthma	Asthma	[50]
circ_0012919	CD4^+^ T cell	increased DNMT1 expression, reduced CD70 and CDlla expression, rescued the DNA hypomethylation of CD1 la and CD70 in CD4^+^T cells of SLE patients, as well as regulated the expression of RANTES and KLF13 by onding to miR-125a-3p	SLE	[51]
circ-LAMP1	T cell	promoting T-cell lymphoblastic lymphoma progression via acting as a ceRNA for miR-615-5p to regulate DDR2 expression.	Lymphoblastic lymphoma	[52]
–	T cell	structure and degradation of circRNAs regulating PKR Activation in innate immunity	–	[53]
circ-CDR1as/ciRS-7	CD8^+^ T cell NK cell Macrophage	regulating the TGF-β signaling pathway and ECM-receptor interaction to serve as a mediator in alteration of the tumor microenvironment	Cancer	[54]
circ-UBAP2	Macrophage Treg Exhausted T cells	mediated ceRNA network modulating PAAD by regulating the infiltration and function of immune cells	PAAD	[55]
circ-PAX5	T cells B cells	up-regulation in pediatric B-precursor acute lymphoblastic leukemia, and disclosed circRNAs with variable expression across cytogenetic subtypes	Pediatric B-precursor acute lymphoblastic leukemia	[56]
circ-PVT1				
circ-HIPK3				
circ-ASAP1	Macrophage	circASAP1 promotes HCC cell proliferation and invasion by regulating miR-326/miR-532-5p-MAPK1 signaling and, mediates tumor-associated-macrophage infiltration by regulating the miR-326/miR-532-5p-CSF-1 pathway	HCC	[57]
circ_0043497	Macrophage	circRNAs alterations are involved in human monocyte derived macrophages response to *Mycobacterium tuberculosis* infection.	Mycobacterium tuberculosis infection	[58]
circ_001204				
circ-ANRIL	Macrophage	circANRIL binds to pescadillo homologue 1 (PES1), thereby impairing exonuclease-mediated pre-rRNA processing and ribosome biogenesis in vascular smooth muscle cells and macrophages.	Ribosomal RNA maturation and atherosclerosis	[59]
circ-HECTD1	Macrophage	SiO2-activated macrophages promoted fibroblast proliferation and migration via the circHECTD1/HECTD1 pathway	Silicosis	[60]
circ-CDC42BPA	B cell	disrupting transduction of B cell signaling to induce occurrence of SCID	SCID	[61]
circ-MALAT1	DC	GDF15 induces tolerogenic DCs (Tol-DCs) through inhibition of circ-MALAT1 and the NFκB signaling pathway and up-regulation of IDO	–	[62]
circ-ARSP91	NK cell	increasing the susceptibility of HCC cells to NK cell cytotoxicity by upregulating UL16 binding protein 1 (ULBP1) expression in HCC cells at the mRNA and protein levels	Hepatocellular Carcinoma	[63]
circ_0000175	Neutrophil	significantly differentially expressed in rheumatoid arthritis, and are related to disease activity and severity	Rheumatoid Arthritis	[64]
circ_0008410				
–	Neutrophil	Differentially expressed circRNAs were mainly involved in immune responses, angiogenesis and metabolism in asymptomatic Moyamoya disease	Moyamoya Disease	[65]
circ-CDR1as	B cell	serving as the miR-7 “sponge” to increase expression of PTEN and restrain	SLE	[66]
		**Other immune-related diseases**		
circ_102584	–	as novel non-invasive biomarkers for SLE	SLE	[67]
circ_0057980	–	circ-0057980 serving as the miR-181d “sponge” to suppress the development of RA. circ-0001045 serving as the miR-30a “sponge” to promote the biogenesis of RA	RA	[68]
circ_0001045				
circ_0088088	–	serving as the miR-16 “sponge” to suppress the development of RA	RA	[69]
circ-Atp9b	–	serving as the miR-138 “sponge” to promote extracellular matrix degradation	OA	[70]
circ_0005105	–	promoting extracellular matrix (ECM) degradation by regulating the expression of miR-26a target NAMPT	OA	[71]
circ_0045714	–	regulating extracellular matrix synthesis as well as proliferation and apoptosis of chondrocytes by promoting the expression of miR-193b target gene IGF1R	OA	[72]
circ-IBTK	–	inhibiting DNA demethylation and activation of AKT signaling pathway by binding to miR-29b	SLE	[73]
circ_0045272	–	combining with miR-6127 to target PAX8 and DTX4 to participate in the secretion of interleukin 2 and the negative regulation of apoptosis	SLE	[74]
–	–	circRNAs involved in atrial fibrillation due to rheumatic heart disease through ceRNA mechanism	Rheumatic	[75]
		**Infection related**		
circ_0000479	–	indirectly regulating RIG-I expression by sponging miR-149-5p, hampering viral replication	Hantaan Virus Infection	[76]
circ-FNDC3B	–	promoting MERS-CoV load and their target mRNA expression which modulates various biological pathways, including the mitogen-activated protein kinase (MAPK) and ubiquitination pathways.	MERS-CoV Infection	[77]
circ-CNOT1				
circ_0004812	–	circ_0004812/miR-1287-5p/FSTL1 axis regulated HBV-induced immune suppression	Chronic Hepatitis B Infection	[78]
**circ_002423**	–	the biological functions of genes hosting the circRNAs were enriched in the progression of metabolism and binding	Meningoencephalitis of Teleosts	[79]
**circ_002834**				
**circ_006192**				
**circ_007265**				
**circ_000305**				
**circ_006456**				
–	–	circRNAs participating in the development of bacterial meningitis through circRNA / miRNA / mRNA regulation mechanism	Bacterial Meningitis	[80]
–	–	circRNAs participating in the resistance to the pathogenesis of diarrhea through circRNA / miRNA / mRNA regulation mechanism	Diarrhea (*Escherichia coli* F17)	[81]
circ_001937	–	correlated with tuberculosis severity	Mycobacterium tuberculosis infection	[82]
–	–	peripheral blood mononuclear cell circRNAs are potentially reliable marker molecules in tuberculosis diagnosis	Mycobacterium tuberculosis infection	[83]
circ_0005836	–	significantly down-regulated in the peripheral blood mononuclear cells of active pulmonary tuberculosis compared with health controls, and might serve as a novel potential biomarker for tuberculosis infection	Mycobacterium tuberculosis infection	[84]

Abbreviations:*SCID* Severe combined immunodeficiency, *SLE* Systemic lupus erythematosus, *RA* Rheumatoid arthritis, *OA* Osteoarthritis, *LUAD* Lung adenocarcinoma, *PAAD* Pancreatic adenocarcinoma, *HCC* Hepatocellular Carcinoma

### circRNAs and CD4^+^ T lymphocytes

CD4^+^ T cells, a vital immune cell in the body’s immune mechanism, i.e., the assistant of the immune system, can direct the body against microorganisms (e.g., pathogenic microorganisms). Zhang C et al. isolated CD4^+^ T cells where circRNA microarray study was conducted for screening out circRNA candidates. As indicated from the results, circ_0012919 reduction enriched the expression of DNMT1, decreased the expressions of CD70 and CD11a, and reversed the DNA hypomethylation of CD11a and CD70 in CD4^+^ T cells of systemic lupus erythematosus (SLE); however, it was reversible by DNMT1 reduction [51]. Circ_0005519 was reported to be up-regulated and negatively related to hsa-let-7a-5p expression in CD4^+^ T cells of asthmatic cases. The fraction of exhaled nitric oxide (FeNO) and the peripheral blood eosinophil rate were positively related to circ_0005519 expression in CD4^+^ T cells. Circ_0005519 expressions between CD4^+^ T cells and PBMCs were harmonious in asthmatic cases. From the mechanistical perspective, circ_0005519 might bind to hsa-let-7a-5p and mitigate inhabitation for IL-13/IL-6 in CD4^+^ T cells [50].

### circRNAs and CD8^+^ T lymphocytes

CD8^+^ T cells are the most common T lymphocyte types. Global circRNAs microarray between plasma of cases with HCC with large CD8^+^ tumor-infiltrating lymphocytes (TILs) and small CD8^+^ TILs effectively reported 6 emerging circRNAs exhibiting different expression. To be specific, the expression of circ_0064428 noticeably decreased in HCC patients carrying high CD8^+^ TILs whereas it was up-regulated in those patients with low CD8^+^ TILs. Besides, circ_0064428 was negatively related to cases’ survival, tumor size and metastasis [85]. Wang YH et al. employed circRNA profiling to investigate circRNA-miRNA interactions in aging human CD8^+^ T cell groups, alongside the loss of CD28 expression. According to their study, circ_100783 may impact phosphoprotein-associated functions during CD28-related CD8^+^ T cell aging. The overlapped circ_100783 expression is likely to denote an emerging biomarker in terms of the longitudinal tracking of CD28-related CD8^+^ T cell aging and global immunosenescence [48]. According to Wang J et al., circ_002178 can facilitate PD-L1 expression by sponging miR-34 in lung adenocarcinoma (LUAD) cells for trigger T-cell exhausting process. Note that circ_002178 can be delivered into CD8^+^ T cells to trigger PD1 expression via exosomes [47]. It was identified that the expressions of CXCR4, HIF1A, ZEB1, and SDC1 in pancreatic adenocarcinoma (PAAD) were controlled by circ-UBAP2 and miR-494. The expressions of CXCR4 and ZEB1 were related to the levels of T-regulating cells (Tregs) and consumed T cells in the PAAD tissues. The expressions of CXCR4 and ZEB1 were positively related to those of CTLA-4 and PD-1 [55], indicating the circUBAP2-mediated ceRNA system regulates PAAD by regulating the infiltrating process and functions of CD8^+^T cells .

### circRNAs in macrophage activation

As a vital part of innate immunity, macrophages are critical to host homeostasis and can change host phenotype and function in accordance with different conditions. Macrophages respond to microenvironmental signals with different activating process, covering conventional activating process exhibiting pro-inflammatory phenotypes (i.e., Ml) and polarimetric activation (M2) featuring an anti-inflammatory spectrum [86, 87]. Classically polarized M1 macrophages activate transitional cells by interferon-gamma (IFN-γ) or other microbial products, e.g., limited partners in turn generate pro-inflammatory cytokines at high levels (e.g., tumor necrosis factor-alpha (TNF), leukocytes Interleukin (IL)-12, IL-23, IL-6, IL-1β, and intermediates generate reactive oxygen species and nitrogen at high concentrations [88–90]. M1 macrophages, as induction and effector cells, promote Th1 response and mediate resistance to intracellular parasites and tumor cells. In contrast, IL-4, IL-13 or immune-complex induced activation of M2 macrophages suggested low IL-4, IL-13 production phenotypes and high IL-10, Arg-1, Fizz1 and Mrc-1 levels [91, 92]. Interestingly, Zhang et al. drew the comparison of circRNA expression spectrum source of bone marrow macrophage (BMDMs) under two diverse polarization (M1 interferon gamma and LPS stimulation attributed to macrophages, M2 macrophages triggered by interleukin - 4), and reported 189 circRNAs various expression for M1 and M2 macrophages, significantly demonstrating the real effect of circRNAs on macrophage polarization [93].

Growing evidence showed that circRNAs are vital in combination with macrophages in the advancement of certain diseases, the most common of which is cancer. Circ-ASAP1 was reported to facilitate HCC cell proliferating and invading processes by regulating miR-326/miR-532-5p-MAPK1 signaling and mediate tumor-related-macrophage infiltrating process through the control over the miR-326/miR-532-5p-CSF-1 path. Clinical HCC samples suggested positive associations between circ-ASAP1 expression and levels of CSF-1, MAPK1, and CD68^+^ tumor-related-macrophages; all these could predict patient outcomes [57]. As suggested previously, Circ-UBAP2 also impacted M2 macrophages activation in the PAAD [55]. Zou Y et al. initially conducted a bioinformatics study on circ-CDR1as among 868 cancer samples with RNA-seq datasets of the MiOncoCirc database. Their data strongly suggests that circ-CDR1as may specifically impact immune and stromal cell infiltrating process in tumor tissue, especially those of CD8^+^T cells, Natural killer (NK) cells stimulated, M2 macrophages, cancer-related fibroblasts (CAFs) and endothelial cells. Systematic and overall studies on circ-CDR1as were conducted to shed light on its underlying pro-cancerous system. Circ-CDR1as controls the TGF-β signaling path and ECM-receptor interacting process to mediate the alerting process of the tumor microenvironment [54].

The relationships between circRNAs and macrophages have been reported in non-tumor diseases as well. Chen X et al. aimed to explore the system of the over activating process of osteoclasts that causes bone homeostasis to be deregulated under non-coding RNA regulating process. Patterns of circRNAs under differential expression were determined in non-treated and RANKL^+^ CSF1-treated bone marrow monocyte/macrophage (BMM) cells. They reported that circ_28313 relieves miR-195a-mediated inhabitation on CSF1 through being a ceRNA, thus conducting the modulation of the osteoclast differentiating process in BMM cells [94]. Huang Z et al. characterized circRNAs expression profiles in human monocyte derived macrophages (MDMs) response to Mycobacterium (Mtb) tuberculosis (TB) infection by microarray assay. As indicated in their outcomes, numerous circRNAs exhibited differential expression in human MDMs after Mtb infection. They found that circ_0043497 and circ_0001204 may be effective diagnostic biomarkers for TB, initially evidencing that circRNAs alterations take part in human MDMs response to TB infection and uncover underlying targets to diagnose and treat TB [58]. The LPS-induced cytoplasmic circRNA, mcircRasGEF1B, and integrate mcircRasGEF1B depletion targeted by transcriptomic study with high throughput to expound its function during the cellular response to LPS stimulating process was stressed and knockdown of mcircRasGEF1B causes modified expression of a wide array of genes. The mentioned results broaden the set of described circRNAs in a functional manner and prove the regulating effect of mcircRasGEF1B in immune response during macrophage activating process and protecting process against microbial infections [95]. Circular antisense non-coding RNA in the INK4 locus (circ-ANRIL), undergoing the transcription at a locus of atherosclerotic cardiovascular disease on chromosome 9p21, endows atheroprotection through the modulation of pathways of atherogenesis and the control over ribosomal RNA (rRNA) maturation. Circ-ANRIL binds to pescadillo homologue 1 (PES1), a critical 60S-preribosomal assembly element, thereby adversely impacting exonuclease-mediated pre-rRNA processing and ribosome biogenesis in vascular smooth muscle cells and macrophages. Thus, circ-ANRIL gives rise to nucleolar stress and p53 activating process, thereby leading to the inducting process of apoptosis and inhibiting process of proliferation, i.e., critical cell functions in atherosclerosis. Overall, the mentioned findings report circ-ANRIL as a prototype of a circRNA controlling ribosome biogenesis and endowing atheroprotection, thus indicating that circularization of long non-coding RNAs may protect human from disease and modify RNA function [59].

### circRNAs and other immune cells

This study delved into CircRNA of AR-hindered PABPC1 91 bp (circ-ARSP91), on immune surveillance triggered by NK cells. Circ-ARSP91 can enhance innate immune surveillance through the increase in the cytotoxicity of NK cells, suggesting that circRNA is likely to impact tumor immunity [63]. Gaffo E et al. found circRNA expression in B-cells, T-cells and monocytes of healthy subjects, covering assumed novel circRNA genes. The comparison of expression considered 6228 circRNAs and stressed cell population-specific expression and exon usage patterns. Differential expression was demonstrated by qRT-PCR for circRNAs specific of B-cells (circ-PAX5, circ-AFF3, circ-IL4R, and circ-SETBP1) or T-cells (circ-IKZF1, circ-TNIK, circ-TXK, and circ-FBXW7), and for circRNAs from intronic (circ-BCL2) and intergenic regions with overexpression in lymphocytes. Based on this resource of circRNA expression in mature blood cell groups, striking and generalized enriched expression of circ-PAX5, circ-PVT1 and circ-HIPK3 in pediatric B-precursor acute lymphoblastic leukemia were found in targeted examining process, and circRNAs exhibiting variable expression across cytogenetic subtypes were revealed [56]. Ma Q et al. delved into the circRNA expressing profile of neutrophil transcriptome in cases carrying asymptomatic Moyamoya disease (MMD). The circRNAs exhibiting differential expression primarily took part in immune responses, angiogenesis and metabolism in asymptomatic MMD [65]. GDF15 was expressed in Dendritic cells (DCs). GDF15 knockout facilitated malat-1 circular RNA (circ_Malat 1), immune responsive functions, DC maturation, while activating the nuclear factor kappa B (NF-κB) pathway [62].

### circRNAs in viral and bacterial infections

According to recently conducted research, circRNA expressions were adversely affected in viral infections and circRNAs were likely to be antiviral targets. Hantaan virus (HTNV), common in Asia, induces hemorrhagic fever having renal syndrome (HFRS) that exhibits large mortality in human race. Some differently expressed RNAs (e.g., GBP1, PARP10, CMPK2, RIG-I, miR-411-3p, miR-330-5p, miR-149-5p, and circ_0000479, facilitated or suppressed HTNV replication). Circ_0000479 regulated RIG-I expression in an indirect manner by sponging miR-149-5p, hindering viral replicating process, which expounds the systems underpinning HTNV-host interaction [76]. Zhang X et al. delved into the host ceRNA network variations and biology-related associations of circRNAs in human lung adenocarcinoma epithelial (Calu-3) cells under the infection of the highly pathogenic Middle East respiratory syndrome coronavirus (MERS-CoV). ≥49,337 putative circRNAs were assessed. Of the 7845 genes generating putative circRNAs, 147 (1.9%) of these genes produced ≥30 putative circRNAs, respectively, and took part in a wide range of metabolic, cellular, and biological processes, covering viral infections. Specific siRNA knockdown of two taken DE circRNAs (circ-FNDC3B and circ-CNOT1) noticeably down-regulated MERS-CoV load and their target mRNA expression, thus modulating a wide variety of biological pathways, covering the ubiquitination and mitogen-activated protein kinase (MAPK) pathways. The mentioned results expound the potential host-targeting antiviral strategies for MERS-CoV infection, biological relevance of circRNAs, and ceRNA network perturbations [77]. Extents of circ_0004812 were enriched in chronic hepatitis B (CHB) cases and HBV-infected hepatoma cells. circ_0004812 knockdown enriched the expression of IFN-α and IFN-β in HBV-infected Huh7 cells. Circ_0004812/miR-1287-5p/FSTL1 axis-controlled HBV-induced immune inhibition [78].

Moreover, circRNAs are demonstrated to be involved in bacterial infections. Yang R et al. employed the sequencing method with high throughput to analyze the transcriptional profiles of host circRNAs in primary brain microvascular endothelial cells that respond to meningitic *E. coli*. 308 circRNAs received significant alteration in total, covering 140 enriched and 168 decreased ones. Through clarifying the transcriptional profiles of the host circRNAs taking part in *E. coli* meningitis, it is speculated that the new knowledge in the regulating systems of circRNAs in the progress of bacterial meningitis will help explain the method of preventing and treating bacterial infections [80]. circ_0005836 and circ_0009128 were significantly reduced in the peripheral blood mononuclear cells (PBMCs) of active pulmonary tuberculosis (APTB) in contrast to health controls (HC) [84]. Hu M et al. established circRNA expression profiles of persistent atrial fibrillation(AF) in cases carrying rheumatic heart disease. Circ_19591, circ_19596 and circ_16175 showed interactions to 36, 28, and 18 miRNAs, respectively; miR-29b-1-5p and miR-29b-2-5p displayed associations with 12 reduced circRNAs, respectively, which proposes an emerging perspective on circRNAs that participate part in AF for rheumatic heart disease and then lays a foundation for subsequent studies on the probable effects of circRNAs on AF [75].

## Conclusions and future perspective

CircRNA in the immune system exhibit several functions: a. regulating the differentiation and development of immune cells, as circRNA can interact with critical factors during immune cell differentiation to differentiate targeted cells; b. regulating the activation state of immune cells and maintaining the homeostasis of cells by regulating critical signaling proteins during cell activation; c. regulating disease response, and the occurrence and development of a series of diseases by regulating the relative balance between pathogens and immune cells and the apoptosis of cells. However, the exact system of action of circRNAs should be further studied. At present, the studies mostly employed RNA sequencing technology to discover novel circRNAs. The number of circRNAs identified is relatively large, how to find functional circRNA from it and clarify its regulating system is considered as the difficulty of circRNA study. This study considers that with the deepening of the study, a growing number of circRNAs involved in immune regulation will be identified. As its system of action is elucidated, new systems are also provided for understanding, and new targets are provided in treating autoimmune diseases, inflammatory diseases and cancers.

## Data Availability

No applicable.

## Abbreviations

circRNAs: circular RNA
miRNA: microRNA
RBPs: RNA-binding proteins
Ago: Argonaute protein
ORF: Open reading frame
IRES: Internal ribosome entry site
m6A: N6-methyl adenosine
FTO: Fat mass and obesity associated
RCD: Regulated cell death
ACR: Autophagy-related circular RNA
HCC: Hepatocellular carcinoma
MAZ: MYC-associated zinc finger protein
TG: Triglyceride
PPAR: Peroxisome proliferator-activated receptor
CRC: Colorectal cancer
DCs: Dendritic cells
NKs: Natural killer cells
FeNO: Fraction of exhaled nitric oxide
TILs: Tumor infiltrating lymphocytes
LUAD: Lung adenocarcinoma
Tregs: T-regulatory cells
TNF: Tumor necrosis factor
IL: Leukocytes Interleukin
BMDMs: Bone marrow macrophage
CAFs: Cancer-associated fibroblasts
MDMs: Monocyte derived macrophages
Mtb: Mycobacterium
TB: Tuberculosis
circ-ANRIL: Circular antisense non-coding RNA in the INK4 locus
rRNA: Ribosomal RNA
PES1: Pescadillo homologue 1
circ-ARSP91: CircRNA of AR-suppressed PABPC1 91 bp
MMD: Moyamoya disease
NF-κB: Nuclear factor kappa B
HTNV: Hantaan virus
MERS-CoV: Middle East respiratory syndrome coronavirus
CHB: Chronic hepatitis B
PBMCs: Peripheral blood mononuclear cells
APTB: Active pulmonary tuberculosis
HC: Health controls
AF: Atrial fibrillation
SCID: Severe combined immunodeficiency
SLE: Systemic lupus erythematosus.
OA: Osteoarthritis
PAAD: Pancreatic adenocarcinoma
MAP3K9: Mitogen-activated protein kinase kinase kinase 9
RIG-I: Retinoic acid inducible gene I
FSTL1: Follistatin-related protein 1
IFN: Interferon
CSF: Macrophage colony-stimulating factor
PES1: Pescadillo homologue 1
DNMT1: DNA methyltransferase 1
GDF15: Growth/differentiation factor 15
MAPK1: Mitogen-activated protein kinase 1
CXCR4: C-X-C chemokine receptor type 4
ZEB1: Zinc finger E-box-binding homeobox 1
CTLA-4: Cytotoxic T-lymphocyte protein 4
